# *N*‑Methylquinuclidinium versus *N*,*N*‑Dimethylpiperidinium Cations on Flexible Side Chains in Anion Exchange Membranes

**DOI:** 10.1021/acsmaterialsau.5c00168

**Published:** 2025-11-03

**Authors:** Si Chen, Triet Nguyen Dai Luong, Patric Jannasch

**Affiliations:** Department of Chemistry, 5193 Lund University, P.O. Box 124, SE-22100 Lund, Sweden

**Keywords:** ion exchange membranes, quaternary ammonium, water electrolysis, fuel cells, alkaline stability, ion conductivity

## Abstract

The conductivity and stability of anion exchange membranes (AEMs) may be significantly enhanced by attaching the cations to the polymer backbones via flexible side chains. Here, we have tethered polydimethylfluorene with the bicyclic “cage-like” *N*-methylquinuclidinium (PdF-Qui) and the monocyclic *N*,*N*-dimethylpiperidinium (PdF-Pip) cations, respectively, via flexible side chains, and studied key AEM properties. Morphological investigations revealed efficient ion clustering in both AEMs, with OH^–^ conductivities exceeding 120 mS cm^–1^ at 80 °C. Alkaline stability studies showed no ionic loss or structural changes in PdF-Qui after storage in a 5 M aqueous NaOH solution at 90 °C for 360 h. In contrast, the benchmark PdF-Pip suffered a 7% loss under the same conditions, primarily via Hofmann elimination. This work presents an efficient synthetic strategy to tether *N*-methylquinuclidinium cations to polymers for AEMs combining outstanding alkaline stability, efficient ionic clustering, and high OH^–^ conductivity.

## Graphical abstract

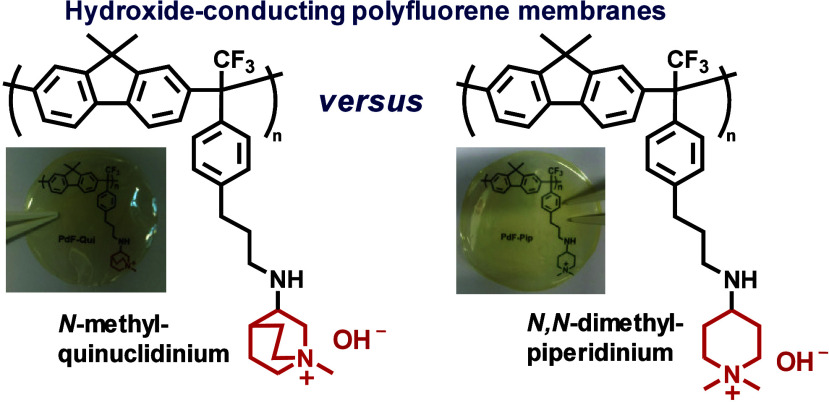

Long-term alkaline stability and OH^–^ conductivity remain the two primary factors limiting the application of AEMs in anion exchange membrane fuel cells (AEMFCs) and water electrolyzers (AEMWEs).^1,2^ Extensive research efforts have recently focused on alkali-stable AEMs based on ether-free polymer backbones and sterically or conformationally hindered cations.^1–11^ Among the studied cations, *N*-cyclic quaternary ammonium cations such as *N*,*N*-dimethylpiperidinium (DMP) and 6-azoniaspiro[5.5]­undecane (ASU) exhibit excellent alkaline stability. Their ring structures bring conformational constraints that hinder ionic loss by Hofmann β-elimination and nucleophilic substitution.^3^ In addition, the stability of the cations is also influenced by their attachment to the backbone polymer.^12–14^ For example, attaching DMP cations directly in a rigid aromatic backbone may increase the distortion and strain of the “soft” aliphatic DMP rings, thus decreasing the alkaline stability. This issue may be alleviated by instead tethering the cations to the backbone via flexible side chains (spacers), which allows the rings to fully relax, thus reducing ring strain and greatly improving the resistance toward Hofmann β-elimination.^12–19^ In addition, the flexible spacers enable local ionic mobility, which generally facilitates ion clustering, thereby enhancing the OH^–^ conductivity of the AEM.^6,14^

Quinuclidiniums are *N*-bicyclic cations with a more rigid structure compared with the monocyclic piperidiniums. The atoms forming the quinuclidine ring are not able to rotate around bond axes and change their relative positions, and the rings are locked in the “boat” conformation. This essentially prevents any strained conformation that would favor Hofmann elimination, which significantly enhances the stability in relation to the more flexible piperidinium ring. Previous studies have reported on quinuclidinium cations tethered to polymer backbones via flexible spacers at the N1 position (e.g., PXQui^20^ in Scheme 1b and PDPF-Qui^12^). These AEMs have demonstrated superior stability compared to their methylpiperidinium (mPip) analogs (e.g., PXmPip^20^), i.e., Hofmann elimination was only observed in the alkyl spacers with no degradation detected in the bicyclic rings under strongly alkaline conditions. Recently, poly­(arylene quinuclidinium)­s with *N*-methylquinuclidinium cations directly attached in the polymer backbone at the C3-position (e.g., PPTQ–OH^21^ in Scheme 1b and PAQ^22^) have been reported to exhibit excellent alkaline stability.^21–27^ No significant structural degradation was detected by NMR spectroscopy after treatment in 10 M NaOH at 80 °C for over 2,500 h.^21–25^

**Scheme 1:**
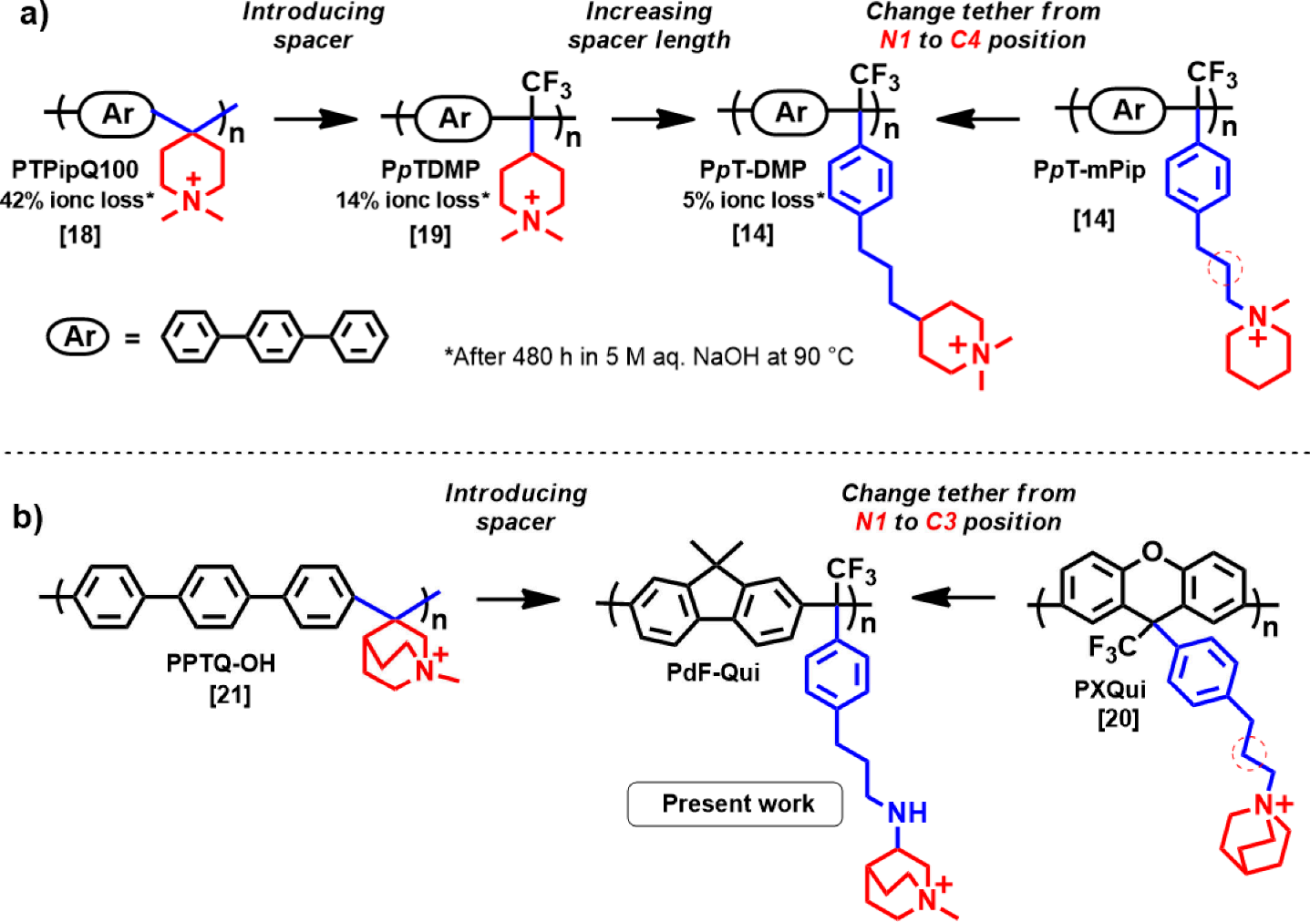
Improving the Performance and Stability of Piperidinium- and Quinuclidinium-Tethered AEMs by Chemical Design^a^

a (a) Previous work on improving the alkaline stability of piperidinium cations in AEMs by introducing and increasing the spacer length and attachment in the C4 position, (b) Present work on quinuclidinium cations attached to polyfluorene in the C3 position via flexible spacers.

Tethering *N*-methylquinuclidinium cations to a polymer backbone via ether-free spacers is more challenging than with corresponding DMP cations due to the general lack of commercially available quinuclidine-containing reactants, e.g., in comparison with corresponding piperidine-containing reactants.^28^ Recently, Hu et al. used 4-amino-*N*-methylpiperidine to prepare AEMs by reacting 3-amino-*N,N*-dimethylpiperidinium (NH_2_-DMP) cations with benzyl chloride groups on a polystyrene backbone.^29^ In the present work, we extended this approach by utilizing 3-aminoquinuclidine to attach 3-amino-*N*-methylquinuclidinium cations to a polyfluorene backbone via a flexible phenylpropyl spacer. A polyhydroxyalkylation involving dimethylfluorene and a bromoalkylated trifluoroacetophenone monomer (TFAp-Br) produced a polyfluorene precursor (PdF-Br, Scheme 2) with a molecular weight of *M*_n_ = 147 kDa and a dispersity of *Đ* = 3.8). The relatively broad dispersity most probably resulted from the final diffusion-controlled stage of the polymerization, when the viscosity was very high. However, because of the high *M*_n_ value, PdF-Br did not contain any low-molecular weight fraction (below ca. 10–20 kDa), which likely would have a negative effect on the mechanical properties (typically causing brittleness) and increasing water uptake and swelling. Hence, the dispersity should have a noticeable but still limited effect on the overall AEM properties. A subsequent base-mediated substitution reaction with 3-amino-*N*-methylquinuclidinium (Scheme 2) functionalized the polyfluorene with quinuclidinium cations (PdF-Qui). In addition, a PdF-Br sample from the same batch was functionalized with piperidinium cations using 3-amino-*N,N*-dimethylpiperidinium. Unlike the symmetry of this cation, the 3-amino-*N*-methylquinuclidinium cation contains a chiral quaternary carbon. Since the physical properties of racemic and enantio-pure polymers may differ significantly,^30,31^ an enantio-pure PdF-sQui was also prepared by reacting PdF-Br with s-3-amino-*N*-methylquinuclidinium to compare with the racemic PdF-Qui. The molecular structures of the polymers were confirmed by ^1^H NMR spectroscopy (Figures S5–S7). For example, signals from the protonated secondary amines in the side chains were observed at approximately 9.0 ppm, and signals from the *N*-methyl groups were observed at 3.0 ppm. Comparisons of the integrated signals for the different aliphatic protons and the aromatic protons indicated complete conversions in the grafting reactions. Additionally, optical rotation measurements indicated the preservation of the chirality of s-3-amino-*N*-methylquinuclidinium in PdF-sQui (Table S1).

**Scheme 2:**
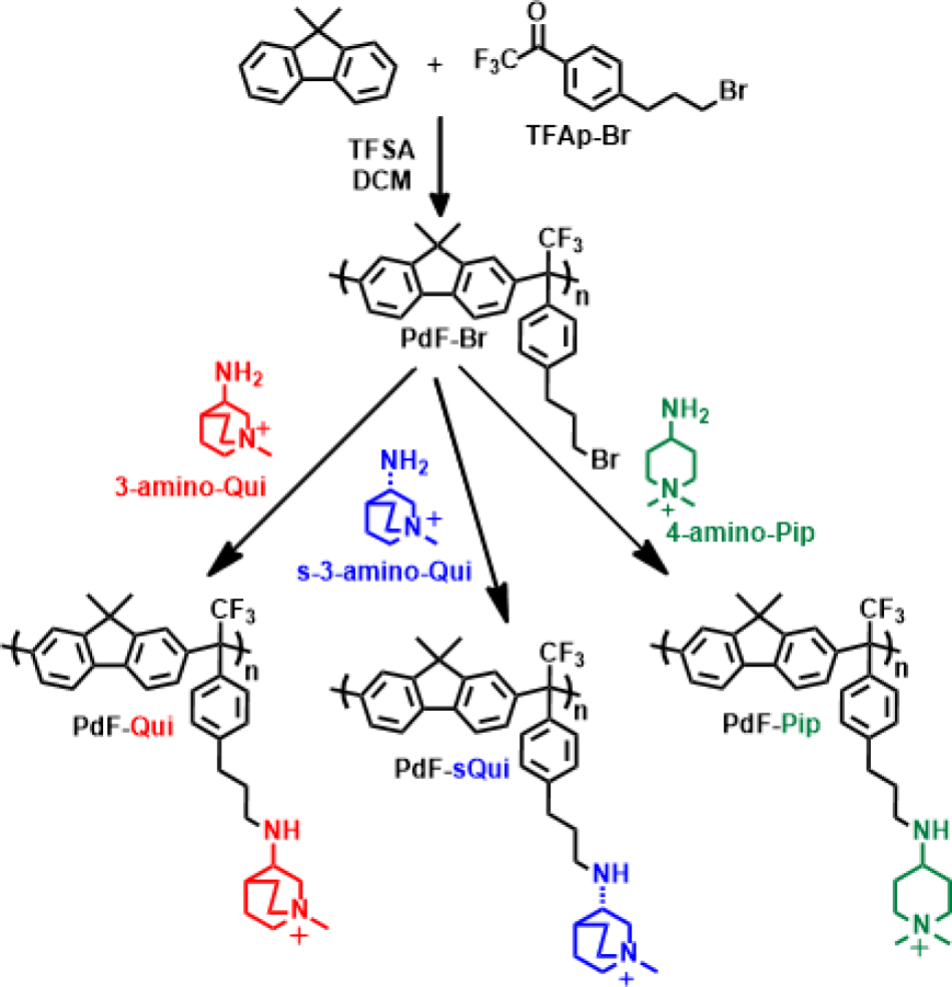
Synthesis of PdF-Qui, PdF-sQui, and PdF-Pip via Polyhydroxyalkylation and Functionalization with Quinuclidinium and Piperidinium Cations, Respectively

AEMs with a thickness of ∼50 μm were cast from dimethyl sulfoxide (DMSO) solutions of the cationic polymers at 48 °C for 3 days. The AEMs were fully transparent and mechanically flexible in the hydrated state. Next, the AEMs were ion-exchanged to the Br^–^ form, and the IEC values were determined by Mohr titrations. As seen in Table 1, there was excellent agreement between the titrated IEC values and the theoretical IECs calculated from NMR data, indicating a complete displacement of the Br atoms on PdF-Br by the cationic moieties. All the AEMs exhibited high thermal stability with thermal decomposition temperatures (*T*_d,95_) above 280 °C (Figure S9). The stress–strain properties of the three AEMs in the Br^–^ form were measured in controlled force mode with a ramping force of 0.8 N min^–1^ at 27 °C (Figure S10). The data of PdF-Qui and PdF-sQui were very similar, with a stress at break of ∼ 35 MPa and a strain at break of 12%, while PdF-Pip showed a stress at break of ∼ 30 MPa and a strain at break of 14%.

**Table 1:** General Membrane Properties

	IEC (mequiv g^–1^)							
AEM	Theoretical^b^	Titrated^c^	WU^a^ (%) (20 °C)	WU^a^ (%) (80 °C)	λ^a^ (80 °C)	σ^a^ (mS cm^–1^) (20 °C)	σ^a^ (mS cm^–1^) (80 °C)	*T*_d,95_ (°C)
PdF-Qui	1.64 (1.82)	1.62 (1.80)	64	89	27	53	122	283
PdF-sQui	1.64 (1.82)	1.63 (1.81)	69	91	28	55	126	281
PdF-Pip	1.67 (1.86)	1.68 (1.87)	61	86	24	45	102	314

a Measured in the OH^–^ form.

b Estimated from NMR data of dissolved AEMs in the Br^–^ form (corresponding calculated IEC of the AEM in the OH^–^ form shown within parentheses).

c Determined by Mohr titration of AEMs in the Br^–^ form (corresponding calculated IEC of the AEMs in the OH^–^ form shown within parentheses).

The morphology of the dry AEMs was characterized by small-angle X-ray scattering (SAXS) and atomic force microscopy (AFM). The SAXS profiles revealed distinct ionomer peaks for PdF-Qui and PdF-sQui at *q* = 2.3 nm^–1^ and for PdF-Pip at *q* = 2.2 nm^–1^, corresponding to *d*-spacings of 2.7 and 2.8 nm, respectively (Figure 1a). The nearly identical SAXS profiles most probably resulted from the very similar IEC values and chemical structures of the AEMs. As seen in Figure 1b-d, the AFM phase images also suggested a high degree of phase separation between hydrophilic and hydrophobic domains for all the membranes. Although the patterns of the hydrophilic phase (dark areas) varied slightly, the dimensions of the hydrophilic phase were found to be in the range 10 – 14 nm for all the AEMs. In comparison, P*p*T-DMP (Scheme 1a) exhibits an irregular morphology and shows no distinct scattering maximum in the SAXS profile.^14^ This suggested that the attachment of the cations via hydrogen-bonding -NH- linkages in the spacer unit facilitated the formation of a quite regular and distinct phase-separated morphology, which may favor ionic conductivity.

**Figure 1:**
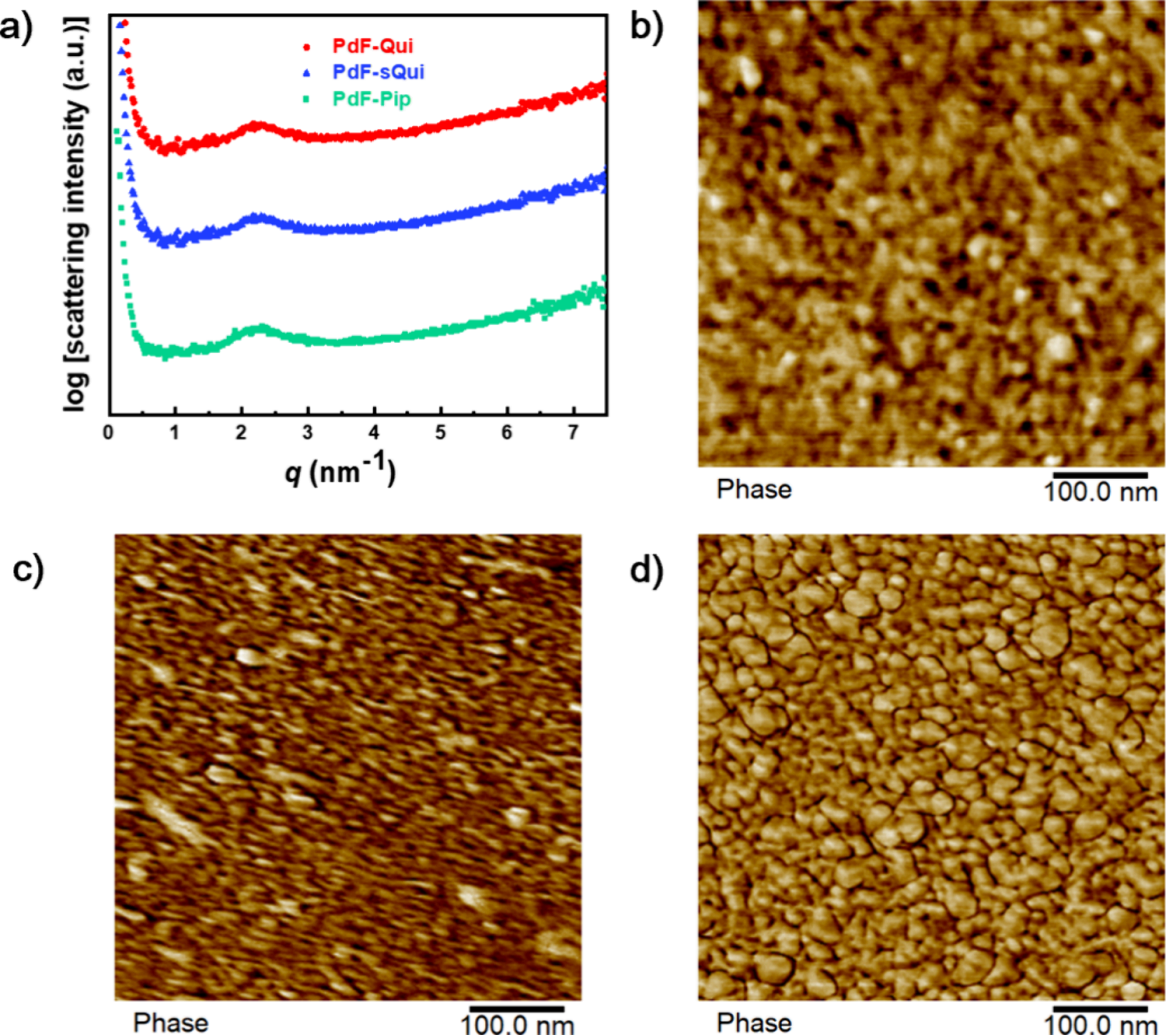
SAXS profiles of the AEMs (a) and AFM phase images of the PdF-Qui (b), PdF-Pip (c), and PdF-sQui (d) membranes.

The water uptake (WU) and swelling ratio (SW) of the AEMs in the OH^–^ form were measured between 20 and 80 °C (Figure 2a-c). Due to the similar IEC values, the WU and SW values reached quite similar levels for the three AEMs. Still, the membranes containing *N*-methylquinuclidinium cations exhibited a slightly higher WU in comparison to the AEM with DMP cations. This difference may be attributed to the stiff and three-dimensional structure of the quinuclidinium cations, which may increase the free volume within the AEM compared to the piperidinium cations, leading to higher WUs.,^12^ The AEMs in this study exhibited WUs ranging from 60 to 70% at 20 °C, significantly higher than the 37% WU previously reported for P*p*T-DMP (Scheme 1 and Figure S8).^14^ Given the similarity of the chemical structure and IEC of P*p*T-DMP, the increased WU and SW of the present AEMs may be attributed to the hydrophilic -NH- linkage in the spacer. The WU increased slowly with temperature. At 80 °C the AEMs reached only moderate WUs (80 – 90%) and SWs (Figure S8), which may result from their modest IEC values (<1.9 mequiv. g^–1^).

**Figure 2:**
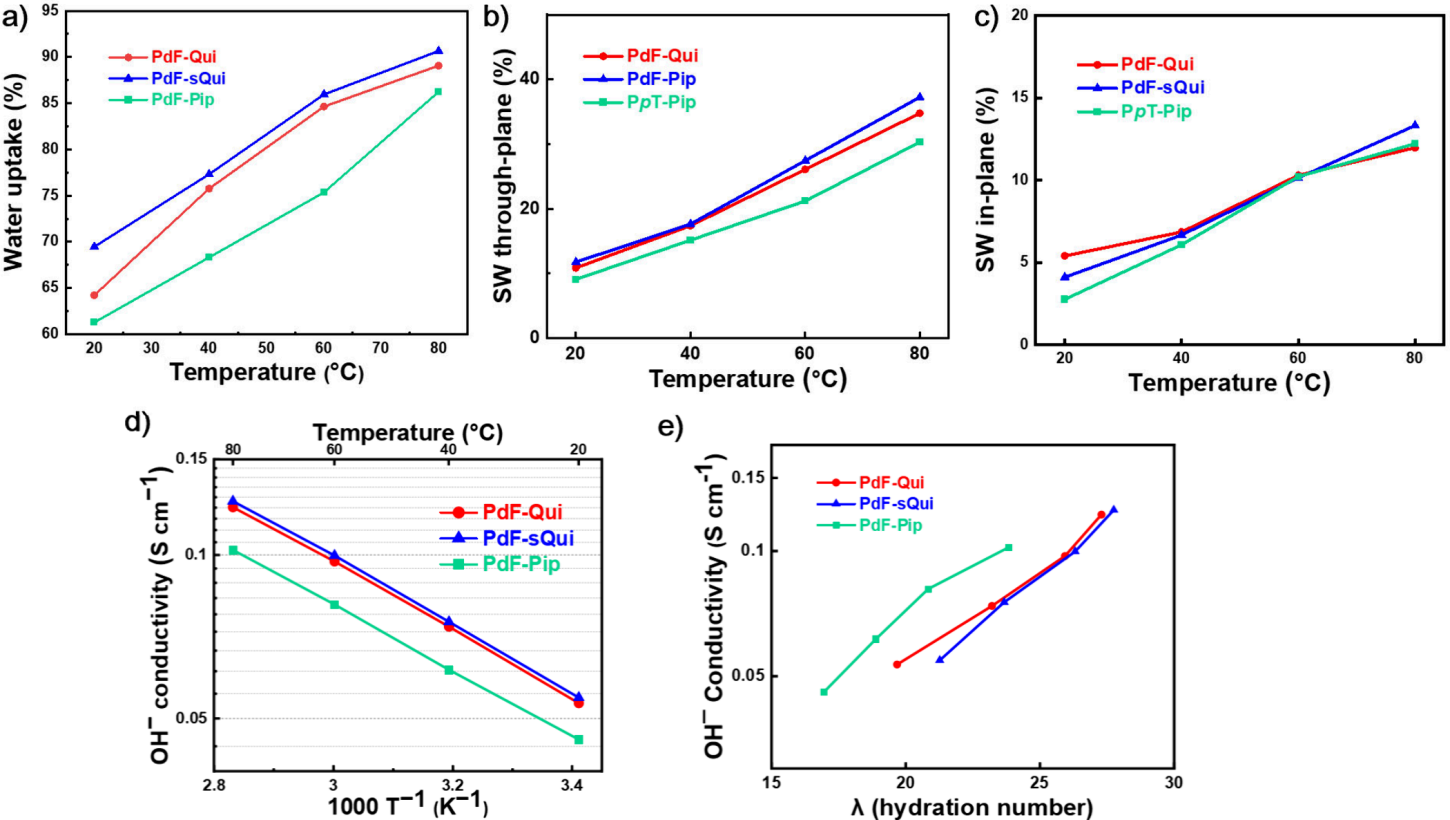
Water uptake (a), and through-plane (b) and in-plane (c) swelling ratios, as well as the OH^–^ conductivity as a function of T^–1^ (d) and the hydration number (e), all measured with the AEMs in the fully hydrated (immersed) state.

The OH^–^ conductivity of the fully hydrated (immersed) AEMs was measured by electrochemical impedance spectroscopy between 20 and 80 °C, and the results followed the WU data of the AEMs. Hence, PdF-Qui and PdF-sQui exhibited higher OH^–^ conductivities than PdF-Pip, reaching above 120 mS cm^–1^ at 80 °C. Membrane PdF-Pip, with a lower WU, showed an OH^–^ conductivity of 102 mS cm^–1^ at the same temperature. As mentioned above, the amine-containing spacer may have promoted both phase separation and hydrophilicity of the AEMs, thereby enhancing the ion conductivity of the AEMs. Consequently, the current AEMs achieved high OH^–^ conductivities despite their limited IEC values.

Designing and preparing highly alkali-stable AEMs were the main motivations of this work. Hence, the alkaline stability of the AEMs was measured after immersion in 5 M NaOH (aq) solution at 90 °C for 360 h. After predetermined storage periods, the samples were ion-exchanged to the Br^–^ form, dried, and dissolved in DMSO-*d*_6_. Trifluoroacetic acid (TFA, 8 vol%) was added before ^1^H NMR analysis to shift the water signal to avoid signal overlap and to protonate any tertiary amines formed after ionic losses due to elimination or substitution reactions. As seen in Figure 3a and b, no significant changes in the spectra recorded before and after the alkaline storage were observed for PdF-Qui and PdF-sQui. In particular, no additional signals appeared between 4 and 6 ppm and between 8 and 10 ppm, respectively, suggesting the absence of any Hofmann β-elimination and nucleophilic substitution reactions causing ionic losses. In contrast, the spectrum of PdF-Pip showed a set of signals at 4.6, 4.8, and 9.7 ppm with a 2:1:1 intensity ratio. This is consistent with the presence of vinylic groups formed by Hofmann β-elimination reactions in the piperidine ring. By comparing the integrals of these signals with the integral of the combined aryl proton signals, the ionic loss was calculated to be approximately 7%. No degradation products from nucleophilic substitution were detected.

**Figure 3:**
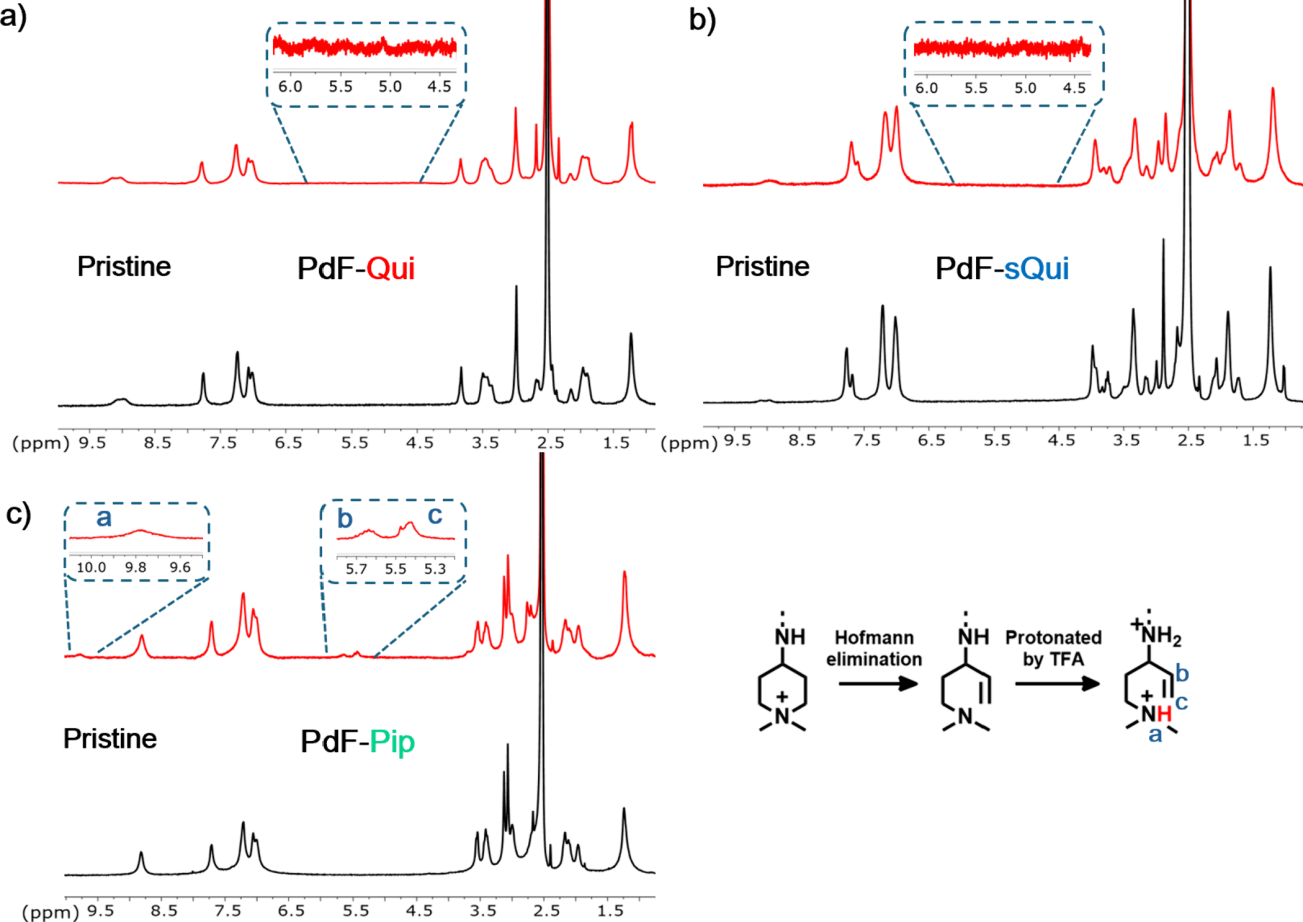
^1^H NMR spectra of (a) PdF-Qui, (b) PdF-sQui, and (c) PdF-Pip before (lower) and after (upper) alkaline treatment during 360 h in 5 M NaOH (aq) at 90 °C (signals a-c, appearing after the treatment of PdF-Pip, indicated Hofmann elimination).

In addition to the direct degradation and loss of the cationic center, the -NH- group in the spacer may potentially be sensitive to the strongly alkaline conditions. This may lead to cleavage of the spacer and the indirect loss of cations. Since the degradation and cleavage of the -NH- group are difficult to detect by NMR, Mohr titrations were employed to compare the IEC before and after the alkaline test. After storage in 5 M NaOH (aq.) solution at 90 °C for 720 h, the IEC values of PdF-Qui and PdF-sQui were measured to be 1.79 and 1.81 mequiv. g^–1^, respectively (Table S2). Hence, compared with the values before alkaline exposure, the IEC values of these membranes remained essentially identical, demonstrating the high stability of the amine-containing spacer and the quinuclidinium cation under harsh alkaline conditions. Testing the alkaline stability under more extreme conditions may provide further insights. However, after immersing the AEMs in 10 M NaOH (aq) solution at 90 °C for 360 h, all the membranes became insoluble, preventing any NMR analysis. Attempts to estimate the ionic loss by Mohr titrations gave non-reproducible results, most probably because of the small AEM samples available from the test. The main reason for the cross-linking reaction was most probably the presence of a small percentage of residual bromoalkyl groups, undetectable by ^1^H NMR spectroscopy and titrations, left after the quaternization of PdF-Br. These groups may form cross-links by direct reaction with the -NH- groups under basic conditions, or be transformed to hydroxyalkyl groups after reaction with hydroxide. The latter groups might, in the deprotonated form, react with the cations in substitution reactions, forming cross-links. Because of the high molecular weight of PdF-Br, only very few cross-links are required to form nonsoluble materials. A similar observation was made in our previous work.^14^ Notably, the ex-situ alkaline stability estimated by this method may not directly reflect the in situ alkaline stability of the AEMs in operating electrochemical devices, but still provides a direct measure of the basic chemical stability.^1,2^

In conclusion, we conveniently tethered *N*-methylquinuclidinium cations and DMP, respectively, to a polyfluorene backbone via a flexible phenylpropylamine spacer. Both the *N*-methylquinuclidinium cations and the spacer demonstrated excellent durability under strong alkaline conditions; no degradation after 360 h storage in 5 M NaOH (aq.) at 90 °C. The hydrophilic amine-containing spacer promoted both phase separation and WU of the AEMs, resulting in OH^–^ conductivities exceeding 120 mS cm^–1^ at 80 °C at a modest IEC of 1.80 mequiv.g^–1^. The properties of the AEMs based on racemic PdF-Qui and enantiopure PdF-sQui were very similar, indicating no significant influence of the stereochemistry. PdF-Pip exhibited both lower WU and OH^–^ conductivity compared to PdF-Qui. Still, PdF-Pip showed distinct ionic clustering and achieved an OH^–^ conductivity of 102 mS cm^–1^ at 80 °C. In contrast to the excellent alkaline stability of the quinuclidinium-functionalized AEMs, PdF-Pip showed ionic losses by Hofmann β-elimination during the alkaline treatment. This work presents a synthetic approach to alkali-stable AEMs by tethering *N*-methylquinuclidinium cations to polyarylenes via flexible and stable spacers. The resulting AEMs showed higher OH^–^ conductivity and superior alkaline stability compared with corresponding AEMs tethered with DMP cations.

## Supplementary Material



## Notes

Experimental descriptions, NMR spectra, specific optical rotation results, TGA traces, and stress–strain data (PDF)
