# Supplementary material for: N‑Methylquinuclidinium versus N,N‑Dimethylpiperidinium Cations on Flexible Side Chains in Anion Exchange Membranes
Source: ACS Mater Au. 2025 Nov 3;6(1):112–8. doi: 10.1021/acsmaterialsau.5c00168 (PMC12810033; doi:10.1021/acsmaterialsau.5c00168)
Supplement: Supplementary file 1 [file mg5c00168_si_001.pdf]

Supporting Information

N-Methylquinuclidinium *versus* *N*,*N*-Dimethylpiperidinium Cations on Flexible Side Chains in Anion Exchange Membranes

Si Chen†*, Triet Nguyen Dai Luong*†*, Patric Jannasch**

Department of Chemistry, Lund University,
P.O. Box 124, SE-22100 Lund, Sweden

1. Materials

Sodium hydroxide (97%, pellets), 3-amino-quiniclidinium (97%), 3-amino-s(-),(-)quinuclidinium (97%), trifluoroacetic anhydride (TFAA, ReagentPlus, 99%), $AlCl_3$ (ReagentPlus, 99%), MeI (98%, stabilized), 1-bromo-3-phenylpropane (98%, Sigma-Aldrich), trifluoroacetic acid (TFA, 99%), Deuterated dimethyl sulfoxide (DMSO-d_6, 99.96 atom% D), K_2CO_3, $NaNO_3$ (99%), and $AgNO_3$ (99%) were all purchased from Sigma Aldrich. triflic acid (TFSA) was obtained from TCI, and anhydrous Na_2SO_4 (99.5%) and NaBr (99%) were purchased from VWR. Diethyl ether (reagent grade), DMAc (99.5%), isopropanol (99.5%), DMSO (99.9%), and ethyl acetate (99.5%) were purchased from Fisher Scientific, and 9,9 dimethyl fluorene (99%) was obtained from AmBeed. Dry dichloromethane for polymerization was collected from an MBraun MB-SPS 800 solvent purification system.

2. Synthesis

2.1 Synthesis of 3-amino-*N,N*-dimethylpiperidinium, 3-amino-*N*-methylquinuclidinium, and 3-amino-s-*N*-methyl-quinuclidinium.

3-amino-*N,N*-dimethylpiperidinium was prepared following a previously reported method.[3]

3-amino-N,N-dimethylpiperidinium: ^{1}H NMR (500 MHz, DMSO-d_6) δ 7.99 (s, 3H), 3.53 – 3.40 (m, 4H), 3.34 (tq, J = 10.7, 5.3 Hz, 1H), 3.07 (d, J = 37.6 Hz, 6H), 2.12 – 1.93 (m, 4H). ^{13}C NMR (126 MHz, DMSO-d_6) δ 59.89, 53.33, 48.87, 44.47, 27.93.

To synthesize 3-amino-*N*-methylquinuclidinium and 3-amino-s-*N*-methyl-quinuclidinium, 8 g (40 mmol) of 3-aminoquinuclidine dihydrochloride or 3-amino-s-quinuclidine dihydrochloride were dissolved in 400 mL of 6 M KOH (aq.) solution in a 1000 mL beaker. After stirring for 10 min, the solution was extracted three times with chloroform (300 mL × 3). The organic phase was collected and dried over sodium sulphate before being concentrated to approximately 40 mL. The resulting solution was then transferred to a 100 mL round-bottom flask. Methyl iodide

(2.3 mL, 5.244 g, 37 mmol) was dissolved in 20 mL of chloroform and added dropwise to the flask via a dropping funnel. After stirring overnight, the resulting yellow solid was collected, dried, and dissolved in 18 mL of DMSO. The DMSO solution was then added dropwise to 120 mL of chloroform, leading to the formation of a white precipitate. The precipitate was collected and dried in a vacuum oven at 50 °C overnight. Approximately 7.8 g of the product was obtained, corresponding to a 72% yield.

3-amino-1-methylquinuclidinium**:** ^{1}H NMR (500 MHz, DMSO-d_6) δ 8.26 (s, 3H), 3.86 – 3.75 (m, 2H), 3.53 – 3.35 (m, 4H), 3.29 (dt, J = 11.8, 3.3 Hz, 1H), 2.98 (s, 3H), 2.28 (q, J = 3.1 Hz, 1H), 2.12 (tdt, J = 10.8, 5.6, 2.9 Hz, 1H), 2.03 – 1.81 (m, 3H). ^{13}C NMR (126 MHz, DMSO-d_6) δ 64.74, 56.40, 55.35, 51.49, 46.29, 26.89 (t, J = 4.0 Hz), 23.02, 17.87.

s-3-amino-1-methylquinuclidinium**:** ^{1}H NMR (500 MHz, DMSO-d_6) δ 8.26 (s, 3H), 3.87 – 3.74 (m, 2H), 3.53 – 3.35 (m, 4H), 3.31 (dt, J = 12.3, 3.5 Hz, 1H), 2.98 (s, 3H), 2.29 (q, J = 3.1 Hz, 1H), 2.13 (tdt, J = 10.9, 5.5, 2.8 Hz, 1H), 2.02 – 1.80 (m, 3H). ^{13}C NMR (126 MHz, DMSO-d_6) δ 64.90, 56.40, 55.36, 51.48, 46.31, 26.92 (t, J = 4.0 Hz), 23.04, 17.88.

2.2 Synthesis of PdF-Br, PdF-Qui, PdF-sQui, and PdF-Pip

The precursor polymer PdF-Br was prepared according to our previously reported procedure.[1,2] In a 25 mL round-bottom flask equipped with a stirring bar in an ice/water bath, TFAp–Br (2.51 g, 8.4 mmol) and dimethyl fluorene (1.5 g, 7.7 mmol) were dissolved in dichloromethane (DCM, 8 mL) under stirring. Next, trifluoromethanesulfonic acid (1.6 mL, 18.1 mmol) was added dropwise to the solution. The reaction proceeded at room temperature for 3 h, before the viscous solution was diluted with chloroform, followed by precipitation of the product in methanol (500 mL). 3.65 g polymer PdF-Br was obtained in a quantitative yield.

To prepare PdF-Qui and PdF-sQui, 1.34 g (5.0 mmol) of 3-amino-methylquinuclidinium and 3-amino-s-1-methylquinuclidinium, respectively, was dissolved in 20 mL of DMAC at 40 °C in a 100 mL round-bottom flask equipped with a stirring Teflon-coated egg. Next, 200 mg (1.45 mmol) K_2CO_3 was added before dropwise adding 240 mg (corresponding to 0.5 mmol repeating units) PdF-Br dissolved in 20 mL of DMAc via a dropping funnel. The reaction was allowed to proceed for 4 days at 48 °C. The reaction mixture was then poured into 400 mL of diethyl ether to obtain a white precipitate. This was collected and washed alternately with isopropanol and water. After drying under vacuum at room temperature, approximately 320 mg of PdF-Qui or PdF-sQui was obtained (96% yield).

PdF-Pip was prepared by a similar method using 3-amino-*N,N*-dimethylpiperidinium dissolved in 30 mL of a DMSO:DMAc (1:1) mixture, and without the use of K_2CO_3.

3. Measurements

3.1 Size exclusion chromatography (SEC)

The molecular weight (M_n) and dispersity ($Đ$) of the precursor PdF-Br polymer were determined by SEC. A Malvern Viscotek instrument was used, equipped with a TGuard (Org Guard Column, 10×4.6 mm) guard column, two PL-Gel Mix-B LS columns (2×30 cm) as

analytical columns, and an OmniSEC refractive index detector. THF was used as eluent at 35 °C at a flow rate of 1 mL min^{-1} during the analysis. A total of eight different polystyrene standards were used for conventional calibration of the SEC data. Six polystyrene standard samples with M_n = 3.6, 15, 470, 650, 2700, 3800 kDa were obtained from Waters Associates (Maple Street, Milford), one sample with M_n = 37 kDa was obtained from Polymersciences Inc., and one with M_n = 10 kDa was obtained from Sigma-Aldrich. The PdF-Br solution (1 mg mL^{-1}) was prepared 24 h before the measurement, and was passed through a PTFE filter (pore size 0.2 μm) before analysis.

3.2 Membrane preparation

The PdF-Qui, PdF-sQui, and PdF-Pip samples were first dissolved in DMSO at a 4 wt% concentration. The solutions were filtered through a PTFE filter (ϕ = 5 μm) before being poured onto a petri dish (ϕ = 6 cm). AEMs were cast during 3 days at 48 °C in an air-circulating oven. Transparent, colorless, and uniform membranes were obtained for all three polymers. The AEMs were subsequently immersed in 3 M NaBr (aq.) for 2 days, before being washed and stored in deionized water (DI water).

3.3 NMR analysis

^{1}H and ^{13}C NMR spectra were recorded using a Bruker DRX 500 spectrometer. Trifluoroacetic acid (TFA) was added when recording ^{1}H NMR (except PdF-Br) to protonate any tertiary and secondary amine groups, and to shift the broad water signal to over 12 ppm, preventing signal overlap. The complete substitution of the Br atoms by the cationic amines (Figure S5-S7) was confirmed by comparing the integral of the methyl group **a** on dimethylfluorene with the sum of signals **b** and **c** in the side chain.

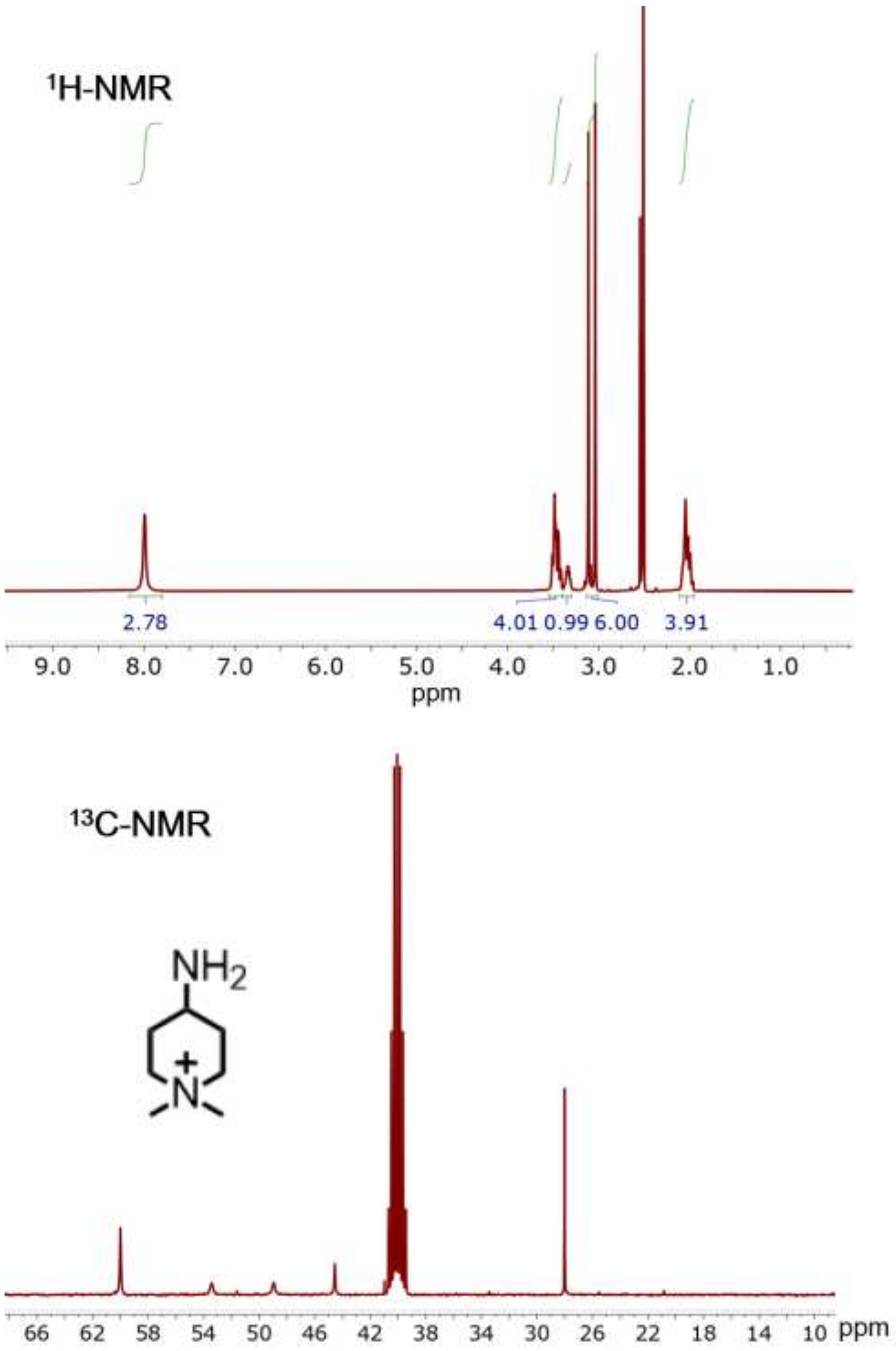

Figure S1. ^{1}H and ^{13}C NMR spectra of 3-amino-*N,N*-dimethylpiperidinium in DMSO-d_6.

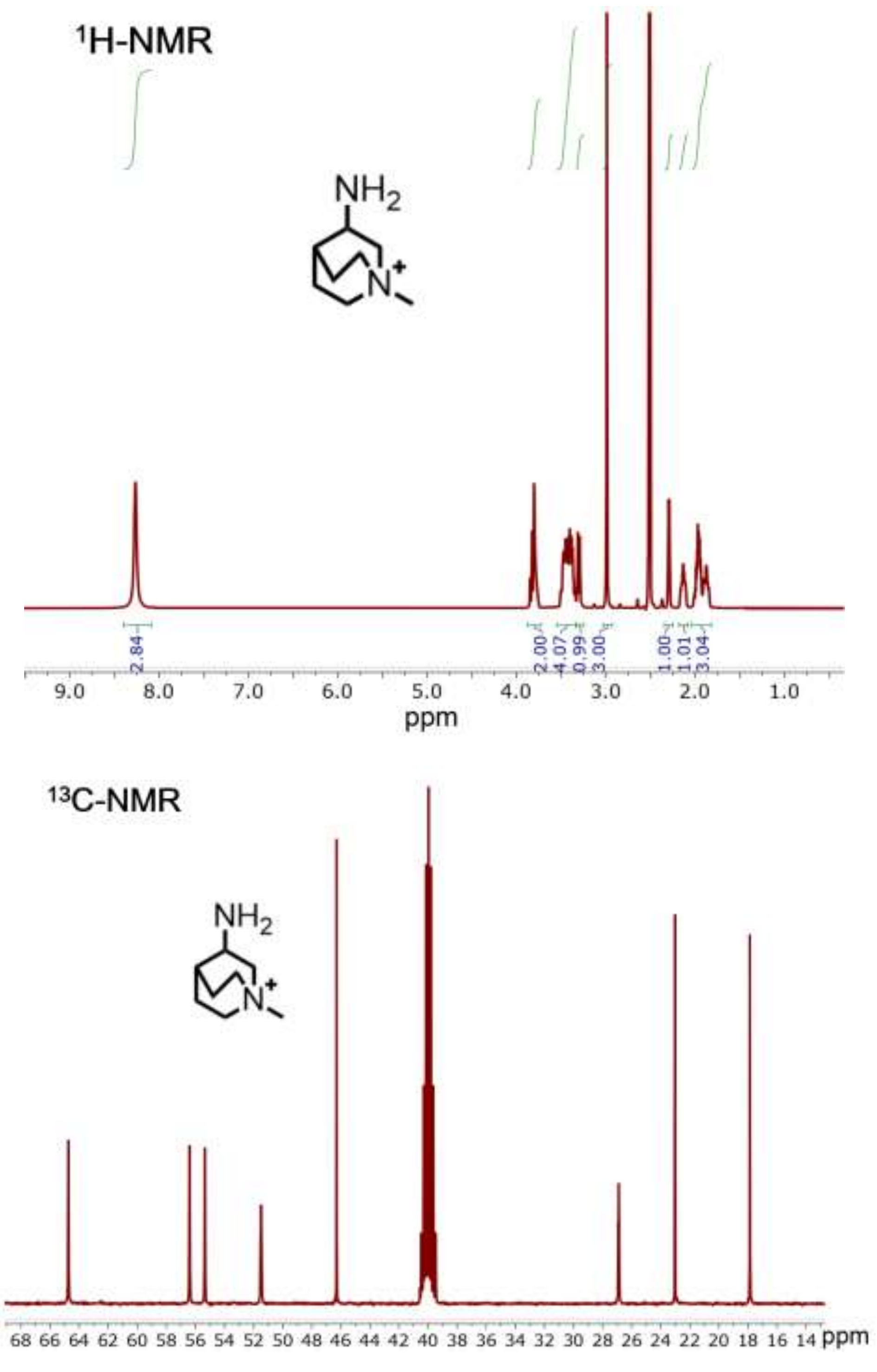

Figure S2. ^{1}H and ^{13}C NMR spectra of 3-amino-*N*-methylquinuclidinium in DMSO-d_6.

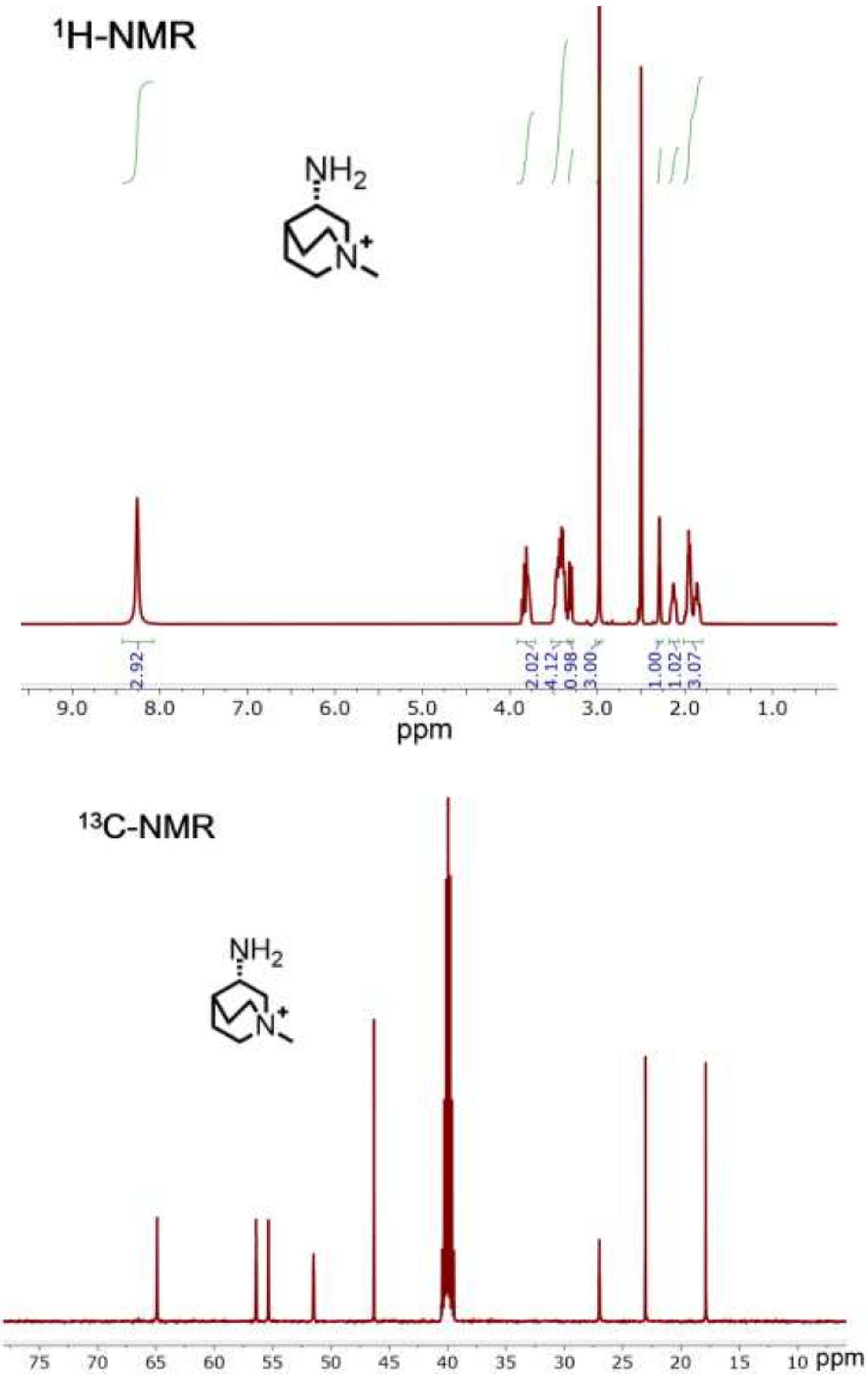

Figure S3. ^{1}H and ^{13}C NMR spectra of 3-amino-s-*N*-methyl-quinuclidinium in DMSO-d_6.

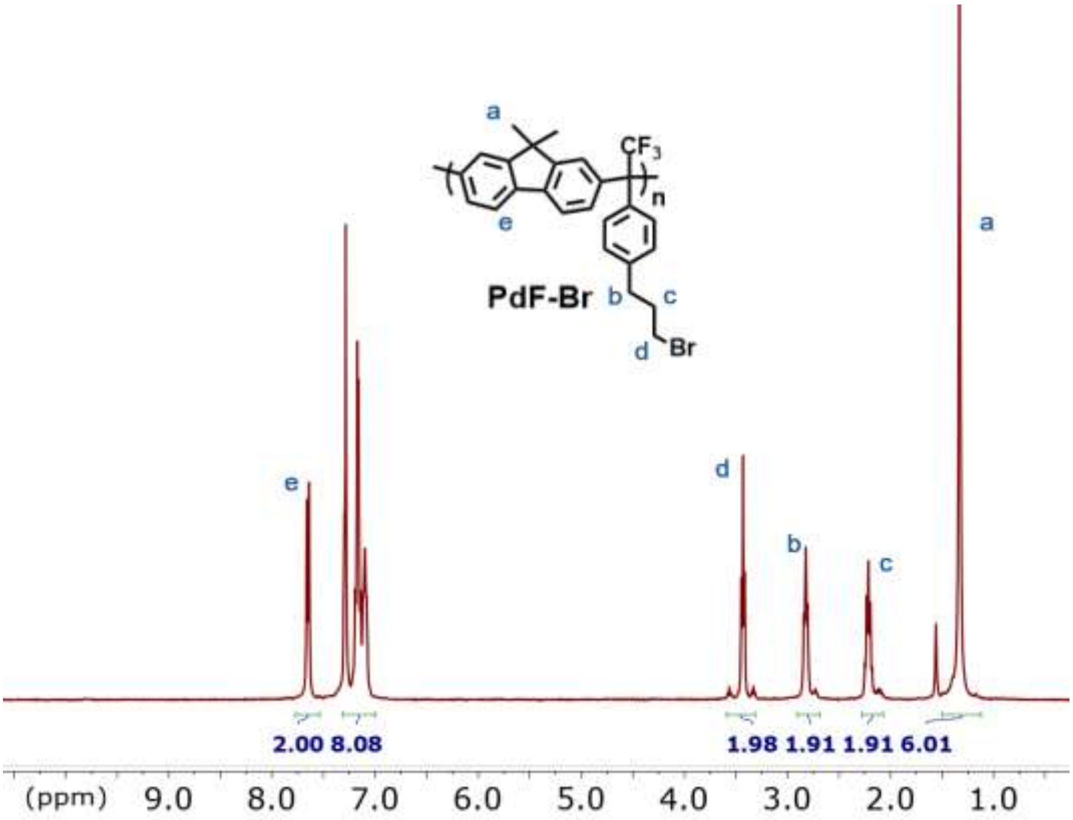

Figure S4. ^{1}H NMR spectrum of PdF-Br in $CDCl_3$-*d*.

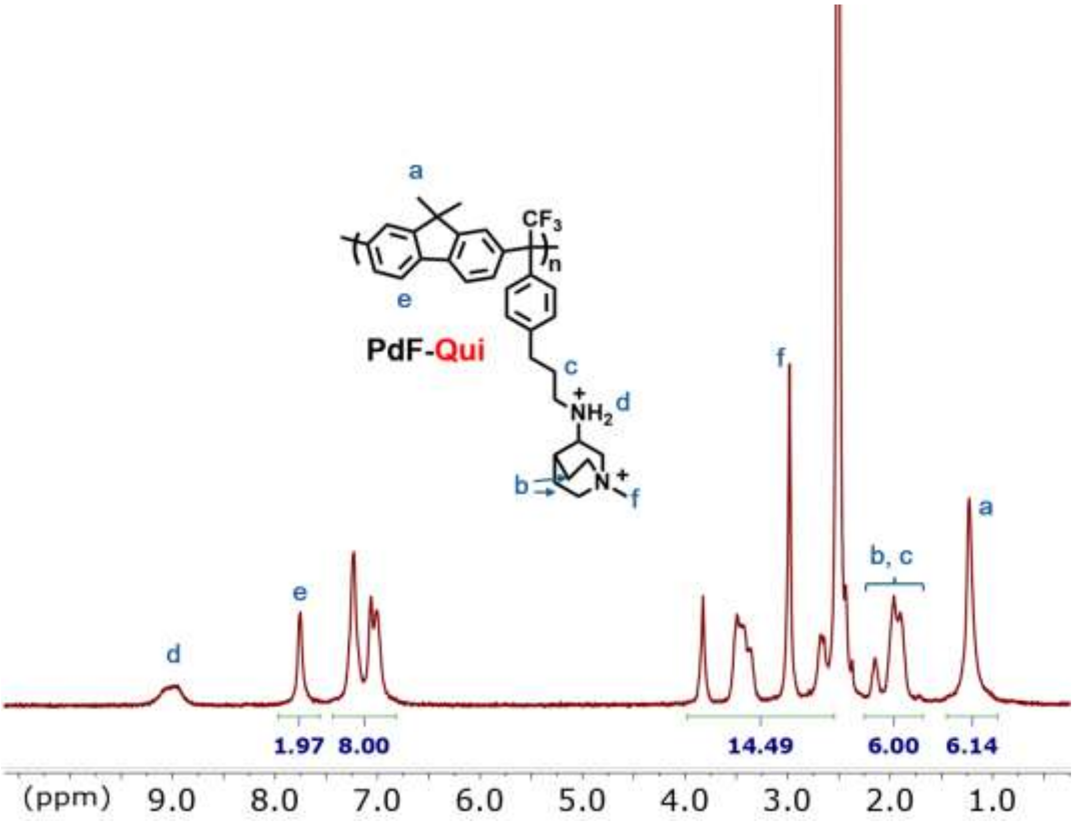

Figure S5. ^{1}H NMR spectrum of PdF-Qui in DMSO-d_6.

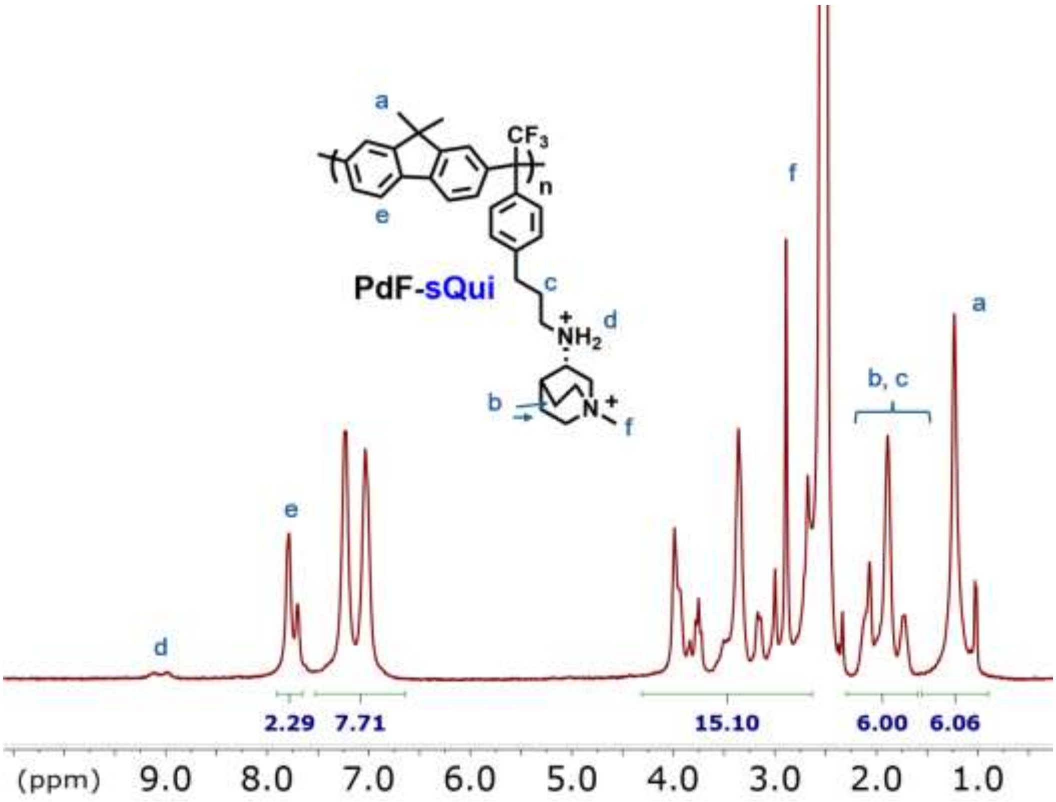

Figure S6. ^{1}H NMR spectrum of PdF-sQui in DMSO-d_6.

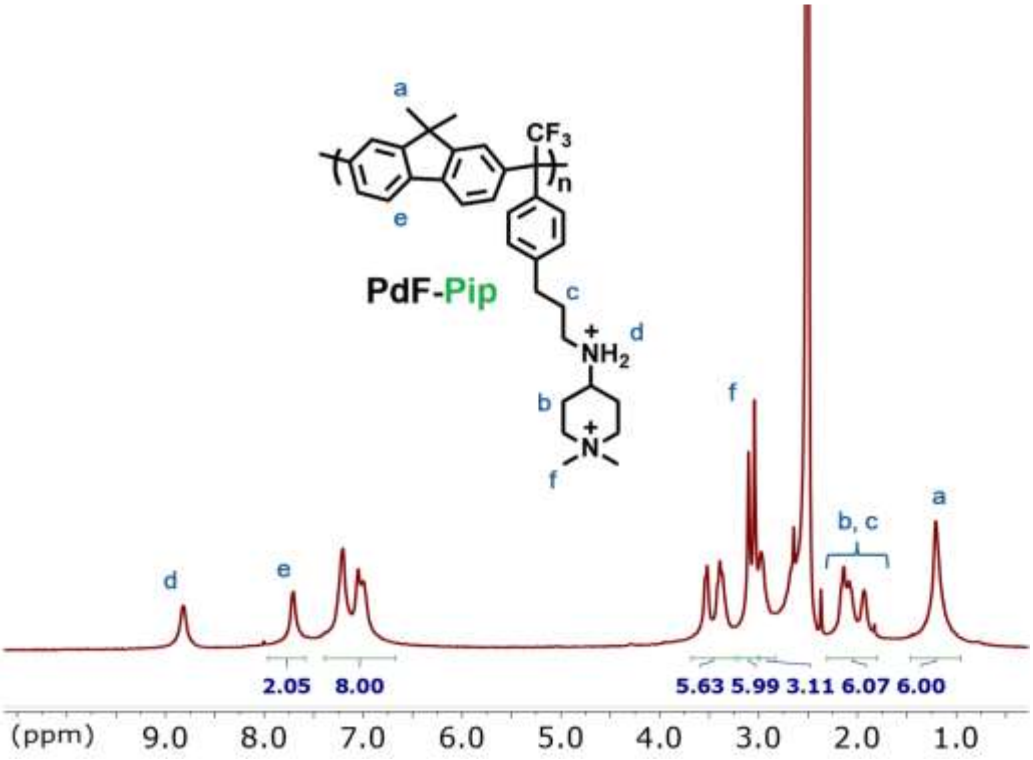

Figure S7. ^{1}H NMR spectrum of PdF-Pip in DMSO-d_6.

3.4 Specific optical rotation

Optical rotations ($[\alpha]_\lambda^T$) were recorded at room temperature (~298 K) using a Perkin Elmer model 341 polarimeter. λ represents the sodium D line (589 nm), and the concentrations (*c*) (0.5 g dL^{-1} in DMSO). The specific rotation was then calculated as:

$$[\alpha]_\lambda^T = \frac{100 \times \alpha}{l \times c}$$ (Eq. S1).

Table S1. Optical rotations of the quinuclidinium compounds.

Compound	$[\alpha]_D^{20}$
3-amino-*N*-methylquinuclidinium	+ 3
3-amino-s-*N*-methylquinuclidinium	- 130
PdF-Qui	+ 1
PdF-sQui	- 12

3.5 Small-angle X-ray scattering (SAXS)

The SAXS measurement was carried out on a SAXLAB instrument (JJ X-ray Systems ApS, Denmark) equipped with a Pilatus detector. Before the measurement, AEM samples in the Br^- form were dried for 48 h. The thickness of the membranes was between 52 and 67 micrometers. SAXS data were collected in the *q*-range 0.14−0.8 nm^{-1}.

3.6 Atomic Force Microscopy (AFM)

A Bruker Icon Atomic Force Microscope instrument with TESPAV2 tips was used in this measurement. Tapping mode was used to record the phase image of AEMs **(**with Br^- as counter ion) in ambient atmosphere.

3.7 Ion exchange capacity, water uptake, and swelling ratio

The ion exchange capacity (IEC) of the AEMs was determined by Mohr titrations. Around 30 mg of each AEMs sample in the Br^- form was dried under vacuum at 50 °C for at least 48 h. These samples were precisely weighed before being immersed in 0.2 M aq. $NaNO_3$ at 40 °C for 2 days. The samples were then re-immersed in fresh 1 M aq. NaBr for 2 days before being washed and immersed in 25 mL 0.2 M aq. $NaNO_3$ at 40 °C for 4 days (note: skipping the second immersion in NaBr solution may lead to an overestimation of IEC). After the completion

of ion exchange, the resulting solution was titrated with 0.01 M aq. $AgNO_3$. K_2CrO_4 was used as a color indicator. The titrations were conducted 4 x 5 mL for each sample. The average result of the four measurements was used to calculate IEC as:

$$IEC_{\mathrm{OH}} = \frac{IEC_{\mathrm{Br}}}{1-0.0629\times IEC_{\mathrm{Br}}} \qquad \text{(Eq. s2).}$$

The water uptake of the AEMs in the OH^- form was measured gravimetrically. Dry samples in the Br^- form were weighed ($W_{\mathrm{dry,\ Br}}$) and then ion-exchanged to OH^- form in 1 M aq. NaOH for 48 h. Then the samples were washed and stored in fresh degassed DI water at 20−80 °C. After 8 h equilibration at each temperature, the sample was taken out, quickly wiped with tissue paper, and weighed ($W_{\mathrm{wet,\ OH}}$). Then the uptake was calculated as follows, with $W_{\mathrm{dry,\ OH}}$ acquired using titrated IEC_{Br}:

$$W_{\mathrm{dry,OH}} = W_{\mathrm{dry,Br}} \times (1 - 0.0629 \times IEC_{\mathrm{Br}}) \qquad \text{(Eq. s3),}$$

$$WU = \frac{W_{\mathrm{wet,OH}} - W_{\mathrm{dry,OH}}}{W_{\mathrm{dry,OH}}} \times 100\% \qquad \text{(Eq. s4).}$$

Along with the uptake measurements, dimensional changes in length (*l*) and thickness (*t*) of AEMs were recorded to investigate the in-plane (SW_{i}) and through-plane (SW_{th}) swelling of the AEMs at different temperatures. A micrometer screw gauge and vernier scale were used during the measurement. Swelling ratios were then calculated as:

$$SW_{\mathrm{i}} = \frac{l_{\mathrm{wet}} - l_{\mathrm{dry}}}{l_{\mathrm{dry}}} \times 100\% \qquad \text{(Eq. s5),}$$

$$SW_{\mathrm{th}} = \frac{t_{\mathrm{wet}} - t_{\mathrm{dry}}}{t_{\mathrm{dry}}} \times 100\% \qquad \text{(Eq. s6).}$$

Table S2. Ionic losses after storage in 5 M aq. NaOH solution as estimated from ^{1}H NMR and titration data.

Method	Time (h)	Corresponding ionic loss (%)	
		PdF-Qui	PdF-sQui
^{1}H NMR spectroscopy	360	Not detected	Not detected
Titration (IEC)	720	0.5	0

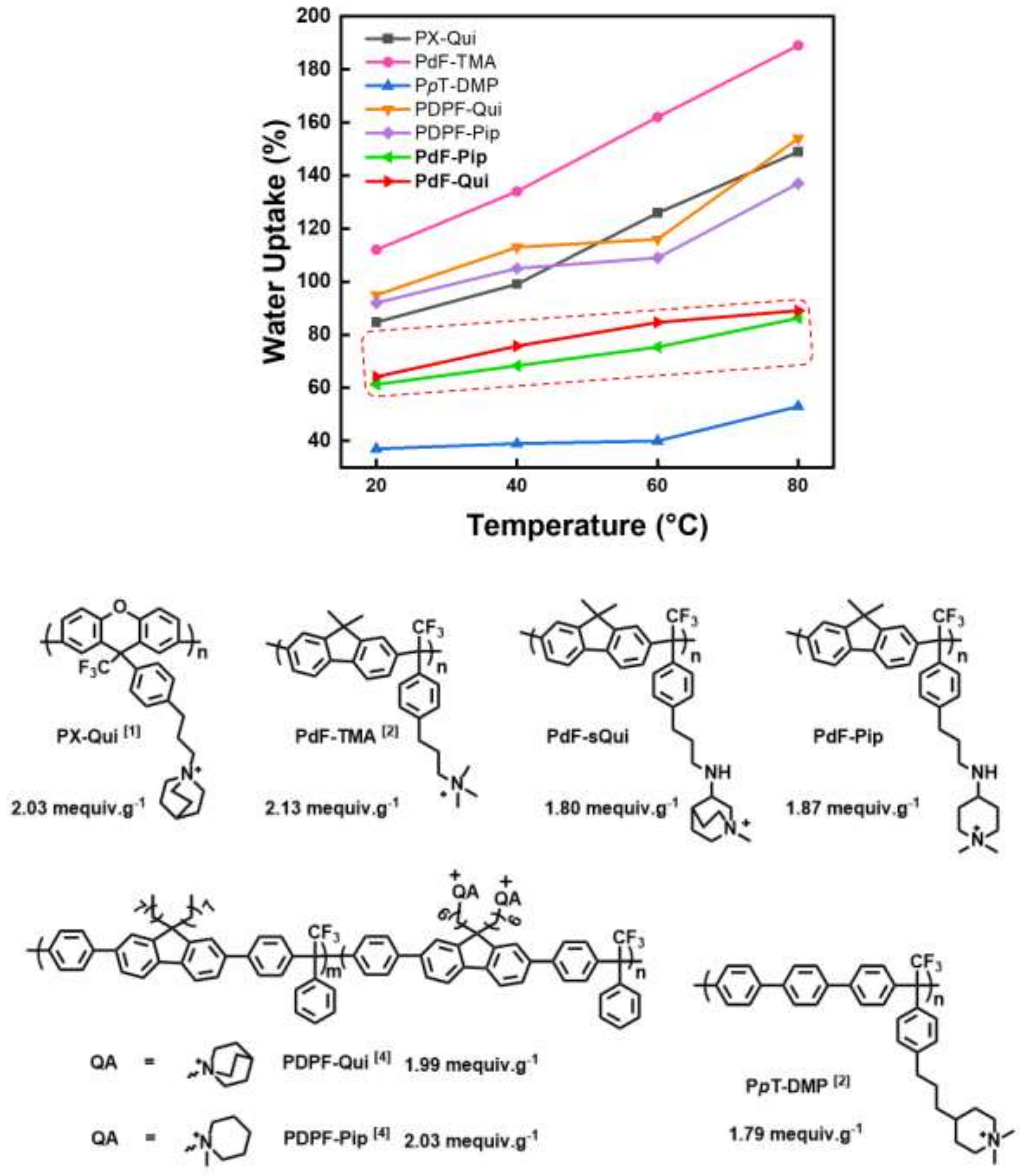

Figure S8. Water uptake versus temperature of the present AEMs and AEMs with similar molecular structures.[1, 2, 4]

3.8 Conductivity measurements

The OH^- conductivity of the fully hydrated AEMs immersed in water was measured by electrochemical impedance spectroscopy (EIS) between 20−80 °C. Square-shaped samples (1.4×1.4 cm) of the AEMs in the Br^- form were prepared and immersed in 1 M NaOH (aq.) at room temperature for 48 h. The alkaline solution was replaced 3 times with a newly prepared NaOH solution during the storage to ensure complete ion exchange. Subsequently, the ion-

exchanged samples were thoroughly washed with degassed DI water and stored in an N_2-ventilated desiccator to prevent CO_2 contamination. The measurements were conducted using a 2-probe cell mounted in a Novocontrol high-resolution dielectric analyzer V 1.01S operating at an amplitude of 10 mV between 10^7−10^0 Hz.

3.9 Thermal stability

The thermal stability of the AEMs in the Br^- form was evaluated by thermogravimetric analysis (TGA) on a TA Instruments TGA Q500 under N_2 atmosphere. Each sample was first preheated at 150 °C for 20 min, before heating from 50 to 600 °C at a rate of 10 °C min^{-1}. The temperature at 5% weight loss was reported as the decomposition temperature ($T_{d,95}$).

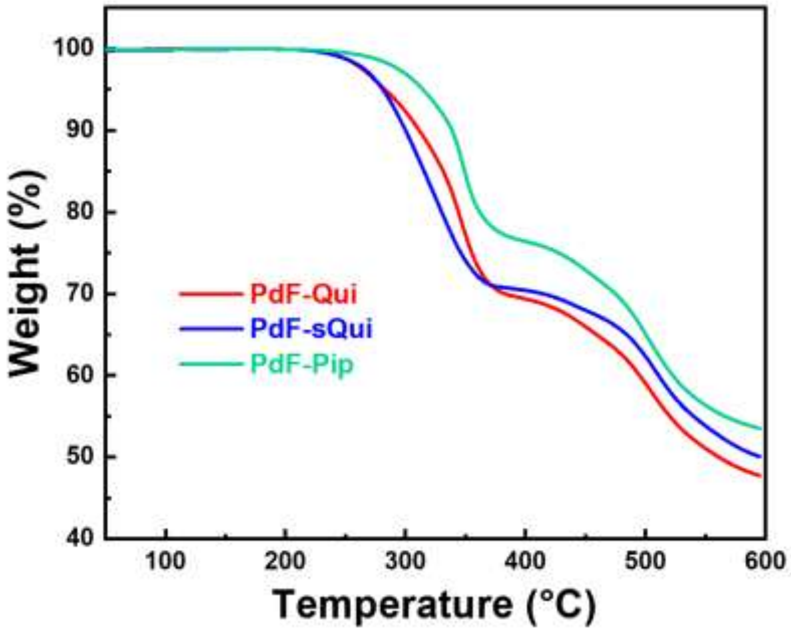

Figure S9. TGA traces of the AEMs, recorded under N_2 atmosphere.

3.10 Alkaline stability

AEM samples were placed in sealed pressure-resistant tubes containing 5 M NaOH (aq.), which were then kept at 90 °C for 360 h. After predetermined storage times, samples were taken out and ion-exchanged to the Br^- form for 48 h, before being washed with DI water and dried. The samples were subsequently analyzed by ^{1}H NMR spectroscopy using DMSO-d_6 as solvent with 5-10 vol% TFA added.

3.11 Mechanical properties

The mechanical properties of the AEMs were evaluated by studying the stress-strain properties of dry AEM samples in the Br^- form using a TA Instruments Q800 dynamic mechanical analysis (DMA). Rectangular samples (approx. 0.4 cm × 2 cm, 60 µm in thickness) were prepared and mounted between the two clamps before applying a 0.05 N preload force.

The measurements were then conducted with a ramping force of 0.8 N min^{-1} at 27 °C in a controlled force mode.

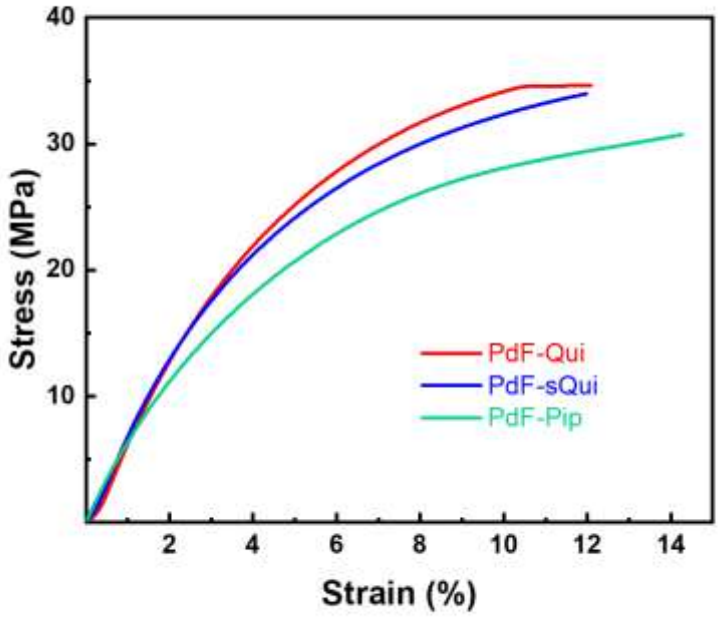

Figure S10. Stress-strain data of the PdF-Qui, PdF-sQui, and PdF-Pip AEMs in the Br^- form recorded in the wet state at 27 °C during a 0.8 N min^{-1} ramping force test.

References

1. Pan, D.; Chen, S.; Jannasch, P. Alkali-Stable Anion Exchange Membranes Based on Poly(xanthene). *ACS Macro Lett.* **2023**, 12, 20– 25,

2. Chen, S.; Pan, D.; Gong, H.; Jannasch, P. Hydroxide conducting membranes with quaternary ammonium cations tethered to poly(arylene alkylene)s via flexible phenylpropyl spacers. *Chem. Mater.* **2024**, 36, 1, 371-381.

3. Chen, N.; Jiang, Q.; Song, F.; Hu, X. Robust piperidinium-enriched polystyrene ionomers for anion exchange membrane fuel cells and water electrolyzers. *ACS Energy Lett.* **2023**, *8*, 10, 4043-4051.

4. Allushi, A.; Pham, T. P.; Olsson, J. S.; Jannasch, P. Ether-free polyfluorenes tethered with quinuclidinium cations as hydroxide exchange membranes. *J. Mater. Chem. A* **2019**, *7*, 27164–27174.
